# A Boolean algebra for genetic variants

**DOI:** 10.1093/bioinformatics/btad001

**Published:** 2023-01-03

**Authors:** Jonathan K Vis, Mark A Santcroos, Walter A Kosters, Jeroen F J Laros

**Affiliations:** Department of Human Genetics, Leiden University Medical Center, 2333 ZC Leiden, The Netherlands; Leiden Institute of Advanced Computer Science, Leiden University, 2333 CA Leiden, The Netherlands; Department of Human Genetics, Leiden University Medical Center, 2333 ZC Leiden, The Netherlands; Department of Clinical Genetics, Leiden University Medical Center, 2333 ZC Leiden, The Netherlands; Leiden Institute of Advanced Computer Science, Leiden University, 2333 CA Leiden, The Netherlands; Department of Human Genetics, Leiden University Medical Center, 2333 ZC Leiden, The Netherlands; National Institute for Public Health and the Environment (RIVM), 3721 MA Bilthoven, The Netherlands

## Abstract

**Motivation:**

Beyond identifying genetic variants, we introduce a set of Boolean relations, which allows for a comprehensive classification of the relations of every pair of variants by taking all minimal alignments into account. We present an efficient algorithm to compute these relations, including a novel way of efficiently computing all minimal alignments within the best theoretical complexity bounds.

**Results:**

We show that these relations are common, and many non-trivial, for variants of the *CFTR* gene in dbSNP. Ultimately, we present an approach for the storing and indexing of variants in the context of a database that enables efficient querying for all these relations.

**Availability and implementation:**

A Python implementation is available at https://github.com/mutalyzer/algebra/tree/v0.2.0 as well as an interface at https://mutalyzer.nl/algebra.

## 1. Introduction

DNA sequencing aims to measure the genetic makeup of individuals. Without going into details about the many different technologies, these processes determine (fragments of) the genetic sequence. Commonly, the primary data analysis consists, among other steps, of (i) alignment against a reference genome, e.g. GRCh38 for human samples and (ii) variant calling. The primary result is a list of variants, i.e. a set of differences, which is specific for the measured individual (sample), often reported in a tabular file like the variant call format (VCF) (Danecek *et al.*, 2011). These variants are used in subsequent applications ranging from fundamental and association research studies to clinical diagnostics. It is advantageous to look only at differences (with regard to some reference), as the genome is usually large (ca. 3 × 10^9^ nucleotides for humans), but the individual differences between two genomes are relatively small [ca. 0.6% (1000 Genomes Project Consortium, 2015)].

When variants are associated with phenotypic traits, they are reported in literature and stored with their annotation in (locus-specific) databases. Usually, the representation of the variant in VCF is refined to a representation more suitable for reporting. For this, many (domain-specific) languages exist. Most notable are:

- Recommendations of the Human Genome Variation Society (HGVS) (den Dunnen *et al.*, 2016);
- SPDI (Holmes *et al.*, 2020), the internal data model for variants used by the National Center for Biotechnology Information (NCBI);
- Variant representation specification (VRS) (Wagner *et al.*, 2021), developed by the Global Alliance for Genomic Health (GA4GH).

These languages attempt to represent the observed differences in a human-understandable and/or machine-interpretable manner and, whereas VCF is implicitly tied to the tooling and configuration used in the primary data analysis, these representations are process agnostic and universally interpretable.

Within the domain of variant recording, some simplifications are common. First, small (local) variants on a single molecular sequence (part of the same haplotype) are recorded separately, because this is convenient when storing large numbers of variants in databases. Phasing information, i.e. whether small variants are part of the same haplotype, is often lost or incomplete. This is partially a direct consequence of the sequencing technology and partially because this information is removed. Second, in some representations (notably, HGVS) uncertainties might be expressed. Usually, the uncertainties relate to the positioning of the variant within the reference genome but also the exact makeup of larger insertions might be unknown. Finally, unchanged regions may be implicit. During primary data analysis, in particular the alignment step, the sequence from the reference genome is assumed to be present even when direct evidence, e.g. coverage information from the sequencing process, is lacking.

For the remainder of this article, we adopt a strict view on the nature of variants:

1. A variant consists of deletions, insertions or a combination thereof with respect to a single molecular sequence. When these operations occur in combination, they are said to be phased, in cis or part of the same allele and can be written down as phase sets or allele descriptions. Many variant description languages have introduced higher-order operations like single nucleotide variants (SNV) (called substitutions in HGVS), multi-nucleotide variants (deletion/insertions), duplications, transpositions, inversions, repeats, etc. We consider all of these notions to be special cases of the definition given above.
2. We consider only *interpretable* variants, i.e. given a *reference sequence*, there is a deterministic and unambiguous way of ‘applying’ the variants such that the result is the (originally) measured *observed sequence*, cf. the Unix diff and patch utilities.

As is already observed within the various variant representation languages, it is often possible to have multiple representations describing the same observed sequence. These possibilities can originate from the choice of ‘operator’, e.g. an SNV can also be represented by a deletion of one nucleotide followed by an insertion of another nucleotide. Another source contributing to the number of possibilities is the structure of the reference sequence. Consider the reference sequence ATTTA and the observed sequence ATTA. One of the symbols T is removed, to say which one specifically yields a number (3) of possibilities. To determine a universally accepted representation of a variant, most variant representation languages employ a *normalization* procedure. Normalization chooses a *canonical* representation from the set of possibilities. Unfortunately, this procedure is not standardized over the various languages, e.g. the 3′-rule in HGVS versus the 5′-rule in VCF. Within a certain language, however, proper normalization solves the problem of identifying *equivalent* variant representations. The implications of using non-normalized variant representations have been reviewed in Yen *et al.* (2017), Eisfeld *et al.* (2019), Pandey *et al.* (2012) and Allot *et al.* (2018). Solutions to this problem are presented in Tan *et al.* (2015), Bayat *et al.* (2017), Watkins *et al.* (2019), Talwalkar *et al.* (2014), Lee *et al.* (2019), Kozanitis *et al.* (2014), Liu *et al.* (2019) and Wittler *et al.* (2015). Often, dedicated tooling (Freeman *et al.*, 2018; Kopanos *et al.*, 2019; Lefter *et al.*, 2021; Vis *et al.*, 2015) is needed to rigorously apply the proposed normalization procedure. Normalized variant representations can be textually compared using standard string matching.

Arguably, identification of equivalent variant representations, i.e. determining whether two variant descriptions result in the same observed sequence, is currently the most interesting query in the variant domain, as it allows for the grouping and matching of equivalent variants and their annotations. With the advent of long-read single molecule sequencing technologies (provided by platforms such as those manufactured by Pacific Biosciences and Oxford Nanopore), which are capable of providing direct evidence of numerous small variants that are part of the same haplotype, a richer set of questions arises. For example, the identification of suballeles, which is of interest in the fields of molecular microbiology (strain typing) and pharmacogenomics (star allele calling), can be achieved by determining whether the suballele of interest is contained within the observed allele.

**Fig. 1.**
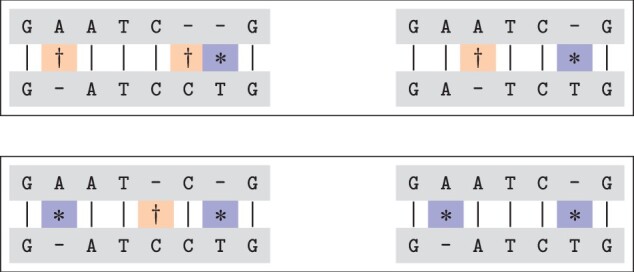
The top panel shows alignments for two variants, GATCCTG and GATCTG, with the same reference sequence GAATCG, where the *changes* (*common to both, ^†^unique for one of them) suggest overlap. The bottom panel shows these same variants, but now obtained through different alignments, where the changes this time suggest that the left variant contains the right one

Minimal sequence-level alignments, informally defined as sequences of deletions/insertions that transform one string into another, having the shortest length possible, are used to define relations between given variants of the same reference sequence. Figure 1 shows an example. In Section 2, we precisely define how the relations depend on the set of all alignments between the two sequences. In the example situation, the containment relation takes precedence over the overlap relation.

In this article, we explore the relations of variants in an exhaustive manner. In addition to the equivalence relation, we partition the domain of binary variant relations into Boolean relations: equivalence; containment, i.e. either a variant is fully contained in another or a variant fully contains another; overlap, i.e. two variants have (at least) one common element; and disjoint, i.e. no common elements. Because of this partitioning, exactly one of the aforementioned relations is true for every pair of variants. For determining the relation, we consider all (minimal) variant representations simultaneously.

## 2. Formalization

Formally, a *variant representation* is a pair (R,φ), where *R* is a *string*, a finite sequence of *symbols* from a non-empty finite *alphabet*, e.g. Σ={A,C,G,T}, called the reference sequence, and φ is a finite set of *operations* transforming the string *R* into the string *O*, the observed sequence. The *length* of a string *S*, denoted by |S|, is the number of symbols in *S*. We refer to the symbol on position *i* of string *S* as *S_i_*, with 1≤i≤|S|. This notation is extended in the natural way for substrings of *S*, i.e. $S_{i\ldots j}$ represents the string containing the contiguous symbols $S_{i},\ldots ,S_{j}$, with 1≤i<j≤|S|.

Note that the set of operations is dependent on the variant representation language used. The actual problem of transforming a reference sequence into an observed sequence is, for instance, handled in Lefter *et al.* (2021).

The difference between the reference sequence (*R*) and the observed sequence (*O*) is the ‘actual’ variant, which is, to some extent, independent from the original representation (φ) as we take *all* minimal representations into account. To this end, we perform a global pairwise alignment between *R* and *O*. In contrast to the specialized alignment methods used in, for instance, the context of short read sequencing, we use an elementary form of alignment which is close to a commonly used distance metric, the *Levenshtein distance* (Levenshtein, 1966). The *simple edit distance*, i.e. the Levenshtein distance without substitutions and weighing both deletions and insertions as 1, is defined as the minimal number *d*(*R*, *O*) of deletions and insertions to transform string *R* into string *O*. It can be determined by d(R,O)=D(|R|,|O|), given by the recurrence relation with 1≤i≤|R| and 1≤j≤|O|:

(1) $$\{\begin{array}{c} \begin{array}{cc} D(0,0) & =0,\\ D(i,0) & =i,\\ D(0,j) & =j,\\ D(i,j) & =\{\begin{array}{cc} D(i-1,j-1) & \text{if}\;R_{i}=O_{j},\\ \min\{\begin{array}{c} D(i-1,j)+1,\\ D(i,j-1)+1 \end{array} & \text{otherwise}. \end{array} \end{array} \end{array}$$

The simple edit distance is related to the *Longest Common Subsequence* (LCS) problem (Bergroth *et al.*, 2000):

(2) $$D(i,j)=i+j-2\cdot |\text{LCS}(R_{1\ldots i},O_{1\ldots j})|.$$

**Fig. 2.**
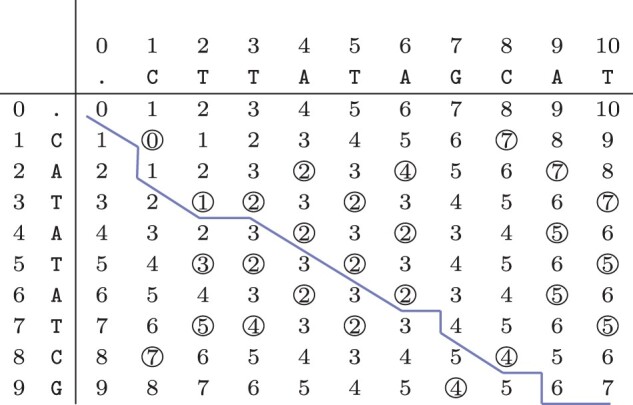
Computation matrix of the simple edit distance between R=CATATATCG and O=CTTATAGCAT. Matching symbols are annotated with a circle. The highlighted path shows one of the minimal alignments

Commonly, the recurrence relation is computed using a dynamic programming approach by filling a matrix containing the solutions to Equation (1) in a bottom-up fashion (Wagner and Fischer, 1974). Consider the computation of the simple edit distance between R=CATATATCG and O=CTTATAGCAT in Figure 2. The simple edit distance D(|R|,|O|)=7 is given by the bottom-right element.

Informally, a representation of string *O* with respect to string *R* (the reference) is a well-defined algorithm to transform *R* into *O*. Formally, it consists of single symbol deletions/insertions (operations) at well-defined string positions from *R*. In the case of insertions, the inserted symbol is also provided; for deletions this is optional. Note that the order of the insertions matters, but deletions can be performed in any order. An easy way to achieve all this is by indexing the positions in *R* (1,2,…,|R|) and providing each operation with the appropriate index from this original numbering. Operations then take place after the position mentioned, where index 0 is used for insertions at the beginning. The ordering issue for insertions can also be resolved by combining the symbols of all insertions at the same position into one string in the desired order.

Note that many languages, like HGVS (den Dunnen *et al.*, 2016), can be used to accomplish the same result. As an example, 8_9insA denotes the insertion of symbol A after the eighth symbol of *R*. Likewise, 7delT represents a deletion of the symbol T at Position 7 of *R*. Together they constitute the representation [7delT;8_9insA], yielding the string O=CATATACAG from reference R=CATATATCG.

A minimal representation is a representation with the smallest number of operations. Such a minimal representation uniquely corresponds to a ‘path’ in the matrix from top-left to bottom-right. These paths can be computed from the matrix by tracing back from the bottom-right element to the top-left element while doing only orthogonal (up or left) steps for non-matching elements if the next element has a lower value than the current one. Vertical steps correspond to deletions, while horizontal steps correspond to insertions. For matching elements (circled) a diagonal step (up and left) is allowed, keeping the current value. Note that matching elements are not recorded in a representation but can easily be inferred: they are exactly the non-deleted positions. For instance, [2delA; 3_4insT; 6_7insG; 7delT; 8_9insA; 9delG; 9_10insT] corresponds to the highlighted minimal representation for the example in Figure 2. Also note that any minimal representation has the same number of deletions and also the same number of insertions.

The computational complexity of the simple edit distance is O(|R|·|O|) (Backurs and Indyk, 2017), although many tailored algorithms exist that have an improved bound for specific classes of strings (Bergroth *et al.*, 2000; Lember *et al.*, 2014; Navarro, 2001; Rick, 2000). In practice, this means that only a subset of the elements in the matrix needs to be computed, in particular if only one solution (or just the distance value) is required.

In general, the number of equivalent trace backs, called LCS *embeddings* in Greenberg (2002, 2003), is exponentially bounded by $\left(\begin{array}{c} \left|R|+|O\right|\\ \left|R\right| \end{array}\right)$. We call the set of all minimal representations Φ(R,O), and we formalize the relations between non-empty variants with regard to a fixed reference sequence *R* (we will omit *R* from our notation for the sake of brevity) by using their respective *O* and *P* observed sequences as generic representations as follows.

Definition 1  (Equivalence) Two variants $\varphi_{O}$ and $\varphi_{P}$ are *equivalent* if and only if Φ(R,O)=Φ(R,P), consequently, *O *=* P*.

**Example:**$R=\mathrm{TTTTTT},\;\varphi_{O}=\mathrm{1delT},\;\varphi_{P}=\mathrm{6delT}$

Here, 1delT (HGVS omits the square brackets in case of a single operation) and 6delT are equivalent because their respective sets of minimal alignments are equal. Classic normalization procedures followed by exact string matching are sufficient to draw the same conclusion. This does not hold for the remaining relations as they rely on checking all combinations of all minimal alignments.

Definition 2  (Containment) The variant $\varphi_{O}$*contains* the variant $\varphi_{P}$ if and only if $\varphi_{O}\prime ⊉\varphi_{P}\prime$ for some $\varphi_{O}^{\prime }\in \Phi (R,O)$ and $\varphi_{P}^{\prime }\in \Phi (R,P)$, and $\varphi_{O}$ is not equivalent to $\varphi_{P}$.

We find a representation within the set of minimal representations for *O* that is a proper subset of a representation within the set of minimal representations for *P*.

**Example:**$R=\mathrm{TTTTTT},\;\varphi_{O}=2\_\mathrm{5delinsGGG},\;\varphi_{P}=\mathrm{3T}>G$2_5delinsGGG (HGVS abbreviation for [2delT; 3delT; 4delT;5delT; 5_6insGGG]) contains 3 T > G (HGVS abbreviation for [3delT; 3_4insG]) and conversely by definition, 3 T > G is contained by 2_5delinsGGG. The containment relation can be easily shown by looking at $\varphi_{O}^{\prime }=[1\_\mathrm{2insG};\mathrm{2delT};2\_\mathrm{3insG};\mathrm{3delT};3\_\mathrm{4insG};\;\mathrm{4delT};\mathrm{5delT}]$ and $\varphi_{P}^{\prime }=[2\_\mathrm{3insG};\mathrm{3delT}]$. All elements of $\varphi_{P}^{\prime }$ are found in $\varphi_{O}^{\prime }$. Different combinations of minimal representations for *O* and *P* possibly yield incomplete results: $\varphi_{O}^{\prime }\prime =[\mathrm{1delT};\mathrm{2delT};3\_\mathrm{4insG};\mathrm{4delT};4\_\mathrm{5insG};\mathrm{5delT};5\_\mathrm{6insG}]$ and $\varphi_{P}^{\prime }\prime =[\mathrm{3delT};3\_\mathrm{4insG}]$, which gives just a single common element (3_4insG), or even $\varphi_{P}^{\prime }\prime \prime =[2\_\mathrm{3insG};\mathrm{6delT}]$ without any common element with $\varphi_{O}^{\prime }\prime$. However, the existence of the combination $\varphi_{O}^{\prime }$ and $\varphi_{P}^{\prime }$ determines the containment relation.

Notable examples of this relation can be found by comparing multiple alleles of polymorphic simple tandem repeats, i.e. a long repeat expansion contains all shorter ones. The variants in Figure 1 are another example of the containment relation.

Definition 3  (Overlap) Two non-equivalent variants $\varphi_{O}$ and $\varphi_{P}$*overlap* if and only if $\varphi_{O}^{\prime }\cap \varphi_{P}^{\prime }\neq \emptyset$ for some $\varphi_{O}^{\prime }\in \Phi (R,O)$ and $\varphi_{P}^{\prime }\in \Phi (R,P)$ while neither $\varphi_{O}$ contains $\varphi_{P}$ nor $\varphi_{P}$ contains $\varphi_{O}$.

A proper subset of a representation within the set of minimal representations for *O* is shared with a proper subset of a representation within the set of minimal representations for *P*.

**Example:**$R=\mathrm{TTTTTT},\;\varphi_{O}=2\_\mathrm{4delinsGG},\;\varphi_{P}=\mathrm{3T}>A$2_4delinsGG has overlap with 3 T > A. A common element (3delT) is easily found: $\varphi_{O}^{\prime }=[1\_\mathrm{2insG};\mathrm{2delT};\mathrm{3delT};3\_\mathrm{4insG};\mathrm{6delT}]$ and $\varphi_{P}^{\prime }=[\mathrm{3delT};3\_\mathrm{4insA}]$, however, the insertion of the symbol A cannot be found in any minimal representation of *O*. Also, the insertion of the symbol G (in *O*) cannot be found in any minimal representation of *P*. In general, the makeup of the common elements, or even the number of common elements between different combinations of minimal representations is not constant.

Polymorphic SNVs are a notable example of the overlap relation, as they share the deleted nucleotide, but the inserted nucleotide is different by definition.

Definition 4  (Disjoint) Two variants $\varphi_{O}$ and $\varphi_{P}$ are *disjoint* if they are not equivalent, are not contained in one another, and do not overlap.

None of the minimal representations of *O* share anything with any of the minimal representations of *P*.

**Example:**$R=\mathrm{TTTTTT},\;\varphi_{O}=2\_\mathrm{3insA},\;\varphi_{P}=4\_\mathrm{5insA}$2_3insA and 4_5insA are disjoint. Although both insert the same symbol (A), this cannot occur at a common position within *R*.

The properties of the Boolean relations given in Table 1 follow directly from the aforementioned definitions. The table is provided for completeness and future reference, and throughout this article, we use these properties to reason about relations.

**Table 1.** Properties of the Boolean relations

Relation	Symmetry	Reflexivity	Transitivity
Equivalent	Symmetric	Reflexive	Transitive
Contains	Asymmetric	Irreflexive	Transitive
Is contained	Asymmetric	Irreflexive	Transitive
Overlap	Symmetric	Irreflexive	Intransitive
Disjoint	Symmetric	Irreflexive	Intransitive

*Note*: The converse of ‘contains’ is ‘is contained’ and vice versa.

## 3. An efficient algorithm

The formal definitions of the Boolean relations presented in Section 2 depend on the enumeration of all minimal variant representations. As explained in Greenberg (2003), the number of representations is bounded exponentially by the length of strings *R* and *O*. For large strings (such as whole human chromosomes up to ca. 250 × 10^6^) this approach is infeasible. In this section, we present an alternative and efficient way for the computation of each of the relations.

**Equivalence**: As follows directly from Definition 1, equivalence can be computed by a string matching over *O* and *P* in O(min(|O|,|P|)) time and O(|O|+|P|) space (storing both strings). This is optimal. Alternatively, we can compute metric *d* for *O* and *P*: d(O,P)=0 if and only if $\varphi_{O}$ is equivalent to $\varphi_{P}$.

**Containment**: We observe that computing the minimal distances is sufficient: d(R,O)−d(R,P)=d(O,P) and d(O,P)>0 if and only if $\varphi_{O}$ contains $\varphi_{P}$. Indeed, in this situation, there is a minimal path from *R* to *O* that passes through *P*, and both legs are minimal too.

**Disjoint**: Again, we note that: d(R,O)+d(R,P)=d(O,P) and d(O,P)>0 implies $\varphi_{O}$ and $\varphi_{P}$ are disjoint, since any minimal paths from *O* to *R* and *R* to *P* are disjoint here. Unfortunately, the converse is not true. Consider the counterexample R=CT, O=TG, and P=GC. *O* and *P* are disjoint despite their simple edit distances being: d(R,O)=2, d(R,P)=2, d(O,P)=2. Their representations, however, have no common elements: Φ(R,O)={[1delC;2_3insG]} and Φ(R,P)={[0_1insG;2delT]}.

The aforementioned distance-based approach can be efficiently computed using any LCS distance algorithm tailored for similar strings, e.g. Wu *et al.* (1990). However, to separate the disjoint and overlap relations, we need to consider all minimal representations. With the notable exception of the naive dynamic programming approach introduced in Section 2, existing algorithms typically do not compute all representations. The naive approach suffers from a O(|R|·|O|) space complexity rendering it infeasible for whole human chromosomes.

### 3.1 Computing all minimal variant representations

Here, we present an efficient algorithm to compute the relevant elements of the recurrence relation (Equation (1)) to be able to reconstruct *all* minimal representations (alignments) within the theoretical complexity bounds: O(|R|·|O|) time and using O(|R|+|O|) temporary space (excluding storing the solution). In practice, because of the high similarity between *R* and *O* the expected run-time is linear. The output of this algorithm is an *LCS-graph* (Rick, 2000): a directed acyclic graph that consists of nodes representing single symbol matches for all LCSs. Edges connect nodes for consecutive symbols in an LCS, possibly labeled with a representation.

We use the generic A* search algorithm (Hart *et al.*, 1968) which uses a heuristic to guide the search. In general, the space requirements of A* search might be of concern. However, in our case, the space is quadratically bounded by the number of elements in the matrix. Furthermore, we demonstrate that by expanding partial solutions in a particular order, it is possible to bound the space requirements linearly: O(|R|+|O|).

We introduce the *admissible heuristic*:

(3) h(R,O,i,j)=|(|R|−i)−(|O|−j)|.

The heuristic *h* represents a best-case guess for the minimal distance from the current element (*i*, *j*) to the bottom-right element of the matrix (hoping to match as many symbols as possible). A* minimizes the total cost function for each solution:

(4) f(R,O,i,j)=D(i,j)+h(R,O,i,j),

by taking into account the actual cost to reach element (*i*, *j*), given by *D*(*i*, *j*) (see Equation (1)), and the estimated minimal cost *h*. A* search iteratively expands partial solutions, also called the *frontier*, based on the lowest *f*-value until the target element is expanded. In our case the progression of *f*-values is determined by the heuristic value of the first element h(R,O,0,0)=||R|−|O||, increasing with steps of 2, as *D* increases by 1 for each orthogonal step and the heuristic changes with either + 1 or −1 for each orthogonal step. Diagonal steps, i.e. matching symbols, do not incur a change in *f*-value. This results in a constant parity for the *f*-values. The simple edit distance is given by the *f*-value of the target element (|R|,|O|). Constructing all minimal variant representations is analogous to the naive approach detailed in Section 2.

In typical A* implementations, the frontier is implemented as a priority queue. In our case, we observe that we can keep track of the elements in the frontier by describing a ‘convex’ shape in the matrix. We use two arrays rows and cols that store the right-most element for a given column and the bottom-most element for a given row, respectively.

**Fig. 3.**
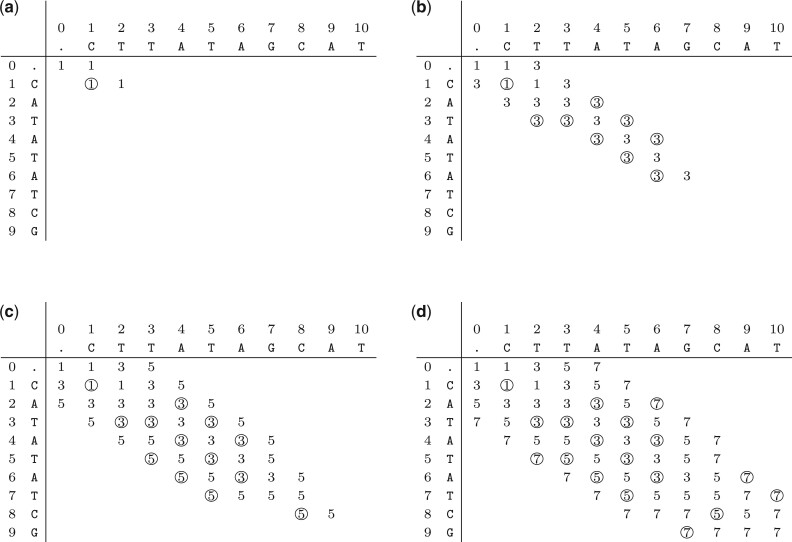
Computing the elements of Equation (4) for R=CATATATCG and O=CTTATAGCAT to efficiently reconstruct the set of all minimal variant representations. (**a**) Expanded elements for *f* = 1 with rows=[1,2] and cols=[0,1,1]. (**b**) Expanded elements for *f* = 3 with rows=[2,3,4,5, 6,6,7] and cols=[1,2,3,3,4,5,6,6]. (**c**) Expanded elements for *f* = 5 with rows=[3,4,5,6, 7,7,8,8,9] and cols=[2,3,4,5,6,7,7,7,8,8]. (**d**) Expanded elements for *f* = 7 with rows=[4,5,6,7, 8,8,9,10,10,10] and cols=[3,4,5,6,7,8,8,9,9,9,9]

In Figure 3, we present the progression of the expansion of the matrix elements for the example introduced in Figure 2: R=CATATATCG and O=CTTATAGCAT. We use O(|R|+|O|) space (excluding the output), and we expand at most O(|R|·|O|) elements.

The non-filled elements are not part of any minimal representation as they would have a greater *f*-value than the bottom-right element. The circled elements are needed to create the LCS-graph and therefore stored. The remaining elements are expanded, but not stored. For each circled element, we determine its place in an LCS (and level in the LCS-graph) by:

(5) $$\left\lfloor \frac{i+j-D(i,j)}{2}\right\rfloor .$$

**Fig. 4.**
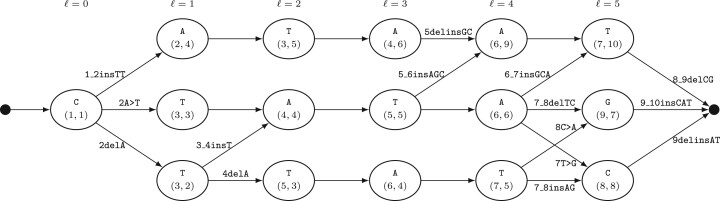
The LCS-graph for R=CATATATCG and O=CTTATAGCAT. The coordinates refer to the coordinates of the matching symbols in Figure 3. Unlabeled edges indicate consecutive matches and do not contribute to the set of elements of all minimal variant representations

This allows us to construct the LCS-graph efficiently. The LCS-graph for the example in Figure 3 is given in Figure 4. The nodes in the LCS-graph are ordered by their position in the LCS. To construct the variant representations, edges are added for each node (*i*, *j*) on level ℓ (determined by Equation (5)) to each node (i′,j′) on level ℓ+1 if i′>i and j′>j. For instance, there is an edge from node (2, 3) on level 1 to node (3, 5) on level 2 (4delA). Not all circled elements end up in the LCS-graph as some do not lie on an optimal path, e.g. T at (2, 5). These elements may be represented as nodes in the LCS-graph. For these nodes there is no path to the sink node. Alternatively, constructing the LCS-graph from the sink node to the source node, these elements are avoided.

We define Ψ(R,O) as the set of all elements that occur in minimal representations from Φ(R,O). To distinguish between the relations disjoint and overlap, it is sufficient to determine whether the two sets Ψ(R,O) and Ψ(R,P) are disjoint. Note that the number of elements in each set is bounded quadratically as opposed to enumerating all, exponentially bounded, minimal representations. Some practical implementation enhancements can also be applied, notably, reducing the number of elements to be added to the set by taking (partially) overlapping edges in the LCS-graph into account. For small alphabets, e.g. DNA nucleotides, an efficient bit string can be used in lieu of a proper set implementation.

### 3.2 Maximal influence interval

Given any pair of variants (within the context of the same reference sequence), it is likely that their relation is disjoint purely based on their often distant positions in the reference sequence. These disjoint relations can be determined efficiently at the cost of some pre-computation for individual variants (n.b. not pairs of variants).

For each variant, the *maximal influence interval* is defined as the interval given by the lowest row index for a deletion or an insertion in an optimal path in *D* and the highest row index for a deletion or an insertion in an optimal path in *D*. This interval gives the extreme bounds, as positions in the reference sequence, of possible changes due to this variant. A pair of variants can only be non-disjoint when their maximal influence intervals intersect. The pre-computing of the maximal influence intervals of individual variants is specifically worthwhile in the context of repeated querying, e.g. a (locus specific) database and VCF annotation.

For example, given a fixed reference R=TCCCTTTA. The variants $\varphi_{O}=\mathrm{3C}>A$ (O=TCACTTTA) with maximal influence interval [2,5) and $\varphi_{P}=\mathrm{6T}>G$ (P=TCCCTGTA) with maximal influence interval [5,8) are disjoint based on the empty intersection of their maximal influence intervals. The variants $\varphi_{O}$ and $\varphi_{P\prime }=[\mathrm{4del};5\_\mathrm{6insC}]$ (P′=TCCTCTTA) with maximal influence interval [2,8) have intersecting intervals, and indeed the variants overlap. In contrast, the variants $\varphi_{O}$ and $\varphi_{P\prime \prime }=2\_\mathrm{3insT}$ (P″=TCTCCTTTA) with maximal influence interval [2,2) also have intersecting intervals, but the variants are ultimately disjoint.

## 4 Experiments

To obtain an intuition of the impact of the proposed approach, we analyzed the well-studied *CFTR* gene (NG_016465.4 with 257 188 bp), that provides instructions for making the cystic fibrosis transmembrane conductance regulator protein.

In dbSNP (build 154) (Sherry *et al.*, 2001), there are 62 215 interpretable variants for the *CFTR* gene which lead to 1 935 322 005 pairs of variants to analyze. Using the method described in Section 3.2, only 92 251 eligible pairs of variants with a potential non-disjoint relation remain.

When the algebra is applied to the remaining pairs, we obtain the results in Table 2. We observe that (as expected) there are no equivalent variants for *CFTR* in dbSNP, indicating a correct application of standard normalization techniques. Beyond equivalence, there are 10 120 containment relations (either contains or is contained), 37 690 pairs have some form of overlap, and 44 441 pairs are disjoint.

**Table 2.** Relation counts for the pairwise comparison of variants in the *CFTR* gene

Relation	Count
Equivalent	0
Contains	5491
Is contained	4629
Overlap	37 690
Disjoint	44 441

*Note*: The counts are given based on the upper triangular matrix, so the converse relations are not included.

Zooming in to individual variant level (as opposed to pairs), we find that 16 939 variants are disjoint with all other variants based on their maximal influence intervals alone and 45 276 variants are potentially involved in a non-disjoint relation with another variant. After determining the relations, 16 814 variants also turn out to be disjoint with all other variants. In total, 33 753 variants are disjoint with all other variants. The remaining 28 462 variants have a non-disjoint relation to some other variant(s).

In Table 3, we see a selection of variants in *CFTR* that, at first sight, have a counter-intuitive relation with another variant. For Pair 1, the left-hand side (LHS) variant contains the right-hand side (RHS) variant because the former can be left justified to 11402_11406del (HGVS abbreviation for the deletion of the symbols on positions 11402,…,11406) to incorporate the deletion of region 11402 to 11403. For Pair 2, the containment is less obvious, the LHS needs to be rewritten to [151240_151241insTATA; 151270_151271insCA] to make this containment relation intuitively clear. For Pair 3, the LHS can be written as [151242_151243del; 151271_151278del] to make the overlap relation between the two variants clear. For Pair 4, left-justification of the LHS to 112270_112271insCTCTCTC and rewriting the RHS to [112269_112270insCC; 112270_112271insCTCT] makes the overlap relation obvious. Finally, we can see from both Pairs 2 and 3 that in practice, variants that are reported to be well separated, still may have something in common.

**Table 3.** Examples of non-trivial relations between variants in *CFTR*

No.	LHS variant	Relation	RHS variant
1	11404_11408del	Contains	11402_11403del
2	151270_151271insTATACA	Contains	151240_151241insAT
3	151271_151280del	Overlap	151240_151255del
4	112274_112275insCTCTCTC	Overlap	112269_112270insCCTCTC

*Note*: The variants are described using the HGVS nomenclature with respect to reference sequence NG_016465.4 using the genomic (g.) coordinate system.

**Fig. 5.**
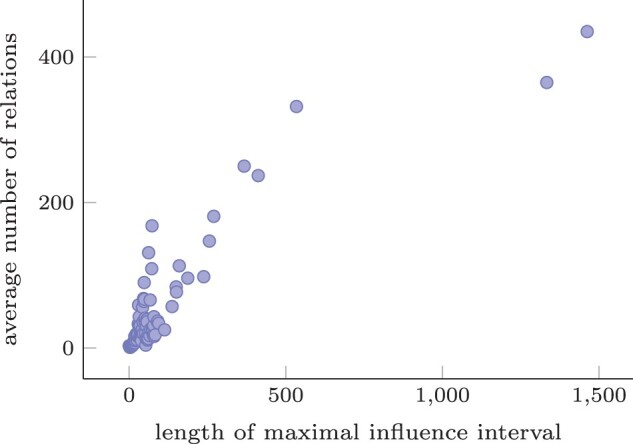
Scatterplot of the average number of non-disjoint relations of all variants in *CFTR* with a certain maximal influence interval length

The ratio between the length of the maximal influence interval and the number of non-disjoint relations a variant has on average is shown in Figure 5. The length of the maximal influence interval correlates strongly with the number of relations of a variant as expected. The variants with the largest maximal influence interval lengths (>150) all happen to be large deletions, e.g. 203907_204783del contains 31 smaller deletions and overlaps with 404 variants.

**Fig. 6.**
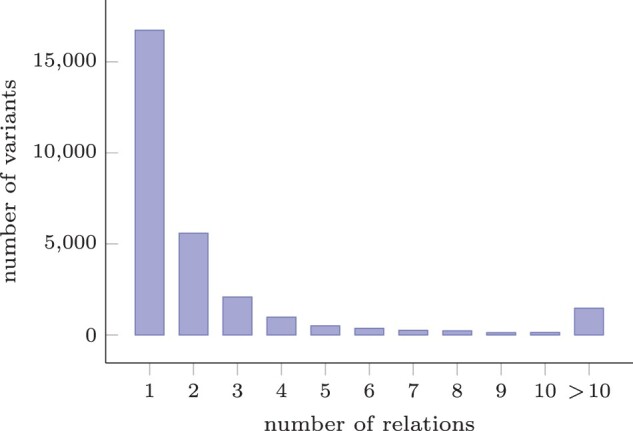
The distribution of the number of non-disjoint relations per variant. The long tail of counts of 11 and above are aggregated. The most relations a single variant has is 435

The distribution of the number of non-disjoint relations per variant is shown in Figure 6. More than half of all variants (16 735) have a single non-trivial relation with another variant, the remaining 11 727 variants have a non-trivial relation with multiple variants. The distributions for both overlap and inclusion relations, are nearly identical.

## 5 Discussion

Higher-order operations like SNVs, multi-nucleotide variants, duplications, transpositions and inversions, can all be represented as combinations of deletions and insertions. In practice, this view aligns well with the expected outcomes, e.g. an SNV can be contained within a larger deletion/insertion. Arguably, inversions are the exception, as their distance represented as a deletion/insertion might not reflect their true nature. This can be considered a limitation of the approach.

The relation between a pair of variants is only well defined when both variants are described in the context of the same reference sequence. In general, we can extend the definitions to include variants on different reference sequences, the natural interpretation of which would be to consider two variants on different reference sequences to be disjoint, e.g. a variant on human chromosome 1 has nothing in common with a variant on human chromosome 2. This interpretation is sensible as long as the reference sequences are unrelated. In practice, however, many reference sequences are actually referring to the same (or a strongly related) genetic locus, e.g. genes on chromosomes, different transcripts for the same gene and chromosomes in different reference genomes. Arguably, variants described in the context of these reference sequences could be seen as having potentially a non-disjoint relation. To properly compare these variants on a sequence level, the differences between the reference sequences should also be taken into account.

Structural variants are often reported in a non-exact manner, i.e. not sequence-level precise. These representations are unsuitable for our method. Even if an exact structural variant representation is given, it is unlikely to yield meaningful results, as the exact positions are not the same across samples. Instead, e.g. gene copies can be analyzed by the algebra when they are provided individually.

The choice of relations presented here follows the ones from set theory, commonly used in a wide range of domains. For some specific domains, more refined relations exists as well, e.g. for intervals, the relations ‘starts with’, ‘ends with’ and ‘is directly adjacent’ are useful extensions (Allen, 1983). The set of relations could be further partitioned using these, or other, refinements.

Unfortunately, the set of relations (see Table 1) does not contain a relation that implies an ordering of variants, i.e. Φ(R,O)≤Φ(R,P). A partial order of variants would require a relation with the following properties: reflexive, antisymmetric and transitive. Sorting variants or storing variants in a particular order in a database (indexing) is meaningless in the context of this algebra. The interval ordering based on the pre-computed maximal influence intervals described in Section 3.2 mitigates this problem.

### 5.1 Characterization of overlap

The actual makeup of the common changes between two variants is never computed. For all relations, except the overlap relation, the common changes can be trivially given: none for disjoint variants, either of the variants for equivalence, and the ‘smaller’ variant for containment, i.e. the one that is contained within the other. This leaves, however, the overlapping variants. In general, there are many different sets of common changes between overlapping variants, some of which, especially the larger ones, may be more (biologically) relevant than others. The algorithm described in Section 3 determines whether there is at least one common change. Computing the maximal size of the overlap requires enumerating an exponential number of possible alignments, which is infeasible for all but extremely short sequences.

### 5.2 General normalization

The current practice of normalizing variant representations is sufficiently powerful to cater for the equivalence relation (also illustrated in Section 4). Determining other relations is, in general, impossible when given a single normalized representation. Even SNVs, often regarded as trivially normalized, are problematic when querying for containment. Consider reference R=CACAT and the SNV 3C>T to obtain the observed sequence O=CATAT. In the classical sense, no normalization is necessary. When we consider a second variant 3_4insT (CACTAT), we might draw the conclusion that this insertion is contained within the SNV based on the normalized position. A possible third variant 2_3insT (CATCAT) has the same relation but is less trivially found. When substrings adjacent to the variant match subsequences of the deleted or inserted string, the number of alignments increases exponentially; therefore, regardless of which normalization procedure is used, however sophisticated, counter examples like this can always be constructed. Therefore, procedures that rely on normalization will, in general, lead to wrong conclusions and cannot be employed to determine relations between variants.

Within the domain-specific languages for variant representations different normalization schemes are used, where arbitrary choices influence the normalized representation, e.g. the 3′ and 5′-rules. From the alignment matrix *D*, it is also possible to choose a canonical path that represents a normalized representation. Sensible choices are either a bottom-most or top-most path. This corresponds to favoring either deletions over insertions at the beginning of a variant (or vice versa). Note that for all minimal variant descriptions in any of the domain-specific languages, corresponding alignments can be found. It could be worthwhile to investigate whether a comprehensive set of deterministic rules exist to find these alignments, as this can be used in the formalization of these languages.

### 5.3 Non-minimal variant representations

So far, we assumed that all variant representations are minimal with regard to Equation (1). In practice, this is not always the case, nor is it necessary for our approach to work, as the only constraint on the variant representation is its interpretability (see Section 1). The relations are computed on all minimal alignments, where a non-minimal representation is minimized as part of the procedure. Interpreting the relations based on non-minimal representations yields surprising results. When we consider the reference R=GCTTT with variant $\varphi_{O}=[\mathrm{1G}>A;\mathrm{2C}>G;\mathrm{3T}>C]$ (O=AGCTT) and variant $\varphi_{P}=[\mathrm{1G}>A;\mathrm{2C}>G]$ (P=AGTTT), the naive conclusion, based on the non-minimal representation, would be that $\varphi_{O}$ contains $\varphi_{P}$. However, both $\varphi_{O}$ and $\varphi_{P}$ are not minimal. The minimal alignments for Φ(R,O)={[0_1insA;3delT],[0_1insA;4delT],[0_1insA;5delT]} and the minimal alignment for Φ(R,P)={[0_1insA;2delC]} show that the actual relation is overlap instead of containment.

A variant representation (in the classical sense) that covers all possible minimal alignments simultaneously is impossible to find in the general case because of the potential mutual exclusivity of subalignments. A trivial solution is the full listing of the observed sequence. This, however, offsets the benefits of a representation that is humanly understandable, and, furthermore, it introduces a huge amount of redundant information for larger sequences. However, based on the maximal influence intervals introduced in Section 3.2, a normalized *supremal* variant representation can be defined. These take the form of a deletion insertion where the deletion spans the entire maximal influence interval and the insertion potentially contains redundant reference information. For the SNV example in Section 5.2, the supremal representation is 2_3delinsAT, where first an A is deleted and inserted again. SPDI (and consequently VRS) prescribes a normalization procedure that follows a similar approach (Holmes *et al.*, 2020) by extending the variant in both directions using a rolling procedure. We note that such a procedure, in general, does not result in all minimal alignments (nor the extreme bounds) being contained in the representation for all variants.

Arguably, a supremal representation is not suitable in all contexts, e.g. reporting clinical results, but within the context of storing large quantities of variants in, for instance a database, the proposed supremal representations are appealing as the variants can be properly ordered and indexed on their deleted interval. Furthermore, these representations contain all information needed to determine the relations with other variants in the database without the need to use the reference sequence. The drawback, however, is that potentially larger inserted sequences are stored (AT in the example). In practice, however, the maximal influence intervals are tiny compared to the length of the reference sequence.

## 6. Conclusions

Looking beyond the identification of equivalent variants, we introduced a comprehensive set of Boolean relations: equivalence, containment, overlap and disjoint, which partitions the domain of binary variant relations. Using these relations, additional variants of interest, i.e. variants with a specific relation to the queried variant can be identified. We determine these relations by taking all minimal alignments (on a sequence level) into account. The relations can be computed efficiently using a novel algorithm that computes all minimal alignments. We have shown that these relations occur frequently in existing datasets, notably in large ones like dbSNP. Approximately half of the variants in the *CFTR* gene in dbSNP have at least one non-disjoint relation with another variant within the same gene. We have shown that normalization of variant representations is not powerful enough to answer any but the trivial relation queries. Inspired by the alignment matrix, we introduced the maximal influence interval of a variant. Filtering on the maximal influence interval allows for calculating the relations of all pairs of variants for an entire gene.

For indexing variants in a database setting, allowing querying on our Boolean relations, we expect that the supremal representation (Section 5.3) will be convenient.

In the case where phased variants (alleles) are available, directly querying on other (combinations of) variants is possible, e.g. is a variant contained within a given allele? The quantification and the makeup of the overlap relation remain an open problem. Locus-specific databases can, without changing their internal representation of variants, use our algebra to query on these relations. Because our method is not tied to a particular representation, it can also be applied in VCF annotation tools.

### 6.1 Future work

The current Python implementation is suitable for sequences up to a length of that of an average gene. Preliminary work on an implementation in a more performance-oriented language indicates that our approach is suitable for handling whole human chromosomes. Although, from the algebra perspective, a single canonical (or normalized) representation is insufficient, we see the advantages of having such a representation in different contexts (especially for human interpretation). By looking at patterns within all the minimal alignments, we can potentially construct a canonical representation that reflects these patterns on sequence level in the variant, e.g. repeated elements can be separated from larger variants or a sequence-level argument can be given for why close by SNVs should be (or not be) combined. These observations could be combined in a new implementation of a variant description extractor (Vis *et al.*, 2015).

Dealing with variants in an algebraic way can possibly be extended to higher-level calculations such as union, subtraction and characterizing/measuring overlap. The ability to mathematically construct larger alleles from smaller variants seems appealing in many domains. These techniques would also enable a proper sequence-level remapping of variants onto other reference sequences, which is a recurring problem with the publication of every new reference genome.

*Financial Support*: none declared.

*Conflict of Interest*: none declared.
